# Use of primary healthcare services prior to suicide in Norway: a descriptive comparison of immigrants and the majority population

**DOI:** 10.1186/s12913-019-4246-3

**Published:** 2019-07-22

**Authors:** Carine Øien-Ødegaard, Anne Reneflot, Lars Johan Hauge

**Affiliations:** Division of mental and physical health, Norwegian Institute of Public Health, PO Box 222, Skøyen, N-0213 Oslo, Norway

**Keywords:** Healthcare use, Suicide, Immigrants, Primary healthcare services, Register data

## Abstract

**Background:**

There is an increase in studies investigating the use of healthcare services prior to suicide. Although studies generally report high usage, there are no previous studies comparing immigrants’ use of primary healthcare (PHC) prior to suicide with that of majority populations. There is a strong influx of immigrants in Europe, and thus a growing demand for filling this knowledge gap and exploiting unused potential for suicide prevention.

**Method:**

By linking three national registers, we examine contact with PHC prior to suicide in all suicide cases in Norway from 2007 to 2014 among individuals aged 15 years and over (*N* = 4341). We report the percentage of individuals in personal contact within the last 6 months, 1 month and 1 week prior to suicide, and use the chi square-test for association.

**Results:**

Overall, immigrants have less contact with PHC prior to suicide. We find significantly lower rates of contact among immigrants, both 6 months and 1 month prior to suicide, for both sexes. The trend is similar in the last week prior to suicide, but less pronounced. The largest variance in contact with PHC prior to suicide is amongst 30–44 year olds. Young, male immigrant suicide victims have the lowest rates of contact with PHC prior to suicide. Contact rates increase with age for all men and women in the majority population, but not for female immigrant suicide victims.

**Conclusions:**

There is a clear difference in rates of contact with PHC prior to suicide between the majority and immigrant populations. The rates are especially low among young males, and measures should be made to lower their threshold for consulting PHC for young males in general and young male immigrants in particular. The difference in contact due to immigrant status appears to be of equal importance as the difference due to sex, although, with few significant results, a conclusion is hard to draw.

## Background

Suicide is one of the main causes of death among young people, and thus a major public health problem [1, 2]. The primary healthcare (PHC) services are an important arena for suicide prevention. PHC is often the first encounter with the healthcare system, and in Norway, the gatekeeper to specialized health services. Receiving adequate treatment at the right time reduces suicide risk [3], but this requires actual contact with healthcare professionals. The suicide prevention potential of a public and accessible PHC services can be leveraged through an awareness of any systematic differences in healthcare utilization. Similarly, knowing who is not in contact prior to suicide, is useful for detecting potential recipients of other preventive measures.

According to two international systematic reviews [4, 5], contact with PHC prior to suicide is common. On average, 80% of suicide victims have been in contact with PHC within the year prior to suicide, 54% within 6 months, and 44% within 1 month. Contact rates vary substantially with age and sex. Both reviews reported higher contact rates among female and older victims. A recent Norwegian study found that 46% of women were in contact with PHC within a month prior to suicide, but only 35% of men [6]. Two studies report the rate of contact within the last week, and found that on average 16% are in contact with their general practitioner (GP) in the last week prior to suicide [7, 8].

Since the second half of the twentieth century, Western Europe has experienced a strong influx of immigrants. Immigrants and Norwegian-born children of immigrant parents now constitute 16% of the Norwegian population [9, 10]. This has sparked an increased demand for knowledge about the use of healthcare by immigrants. A systematic review of suicide among immigrants in Europe found no generalizable pattern [11]. They concluded that immigrants “bring along” the suicide risk of their home country. A recent Norwegian study found a lower suicide rate among immigrants than the majority population, although they did not distinguish suicide rates according to immigrants’ country of origin [12].

In their review, Stene-Larsen and Reneflot noted that no prior studies have examined contact with PHC prior to suicide among immigrant populations [5]. Studies examining immigrants’ use of PHC for mental health problems have found that immigrants are less likely to contact PHC than the majority population [13, 14]. This is unlikely to be explained by lower levels of mental health problems among immigrants than the majority population. Instead, it is suggested that immigrants experience barriers in accessing care for mental health problems [13, 14]. Studies of immigrants’ use of PHC in general show the same pattern [15]. Whether similar patterns exist for PHC use prior to suicide has not yet been determined.

Our main assumption, based on our knowledge of immigrants’ use of PHC both in general and for mental health problems, is that immigrants have a lower contact rate prior to suicide. However, the difference is likely to vary at different points in time prior to suicide. We can surmise that the problems causing the suicide are more serious closer in time to the suicide. On the one hand, this may make the barriers for help seeking greater, resulting in larger differences between the population groups. On the other hand, the increase in problems may also lead to more immigrants seeking help, thus evening out potential differences between the population groups.

In this study, we will investigate whether there are differences in contact patterns at different points in time prior to suicide between immigrants and the majority population of Norway. We use national register data to investigate differences in contact with PHC prior to suicide between immigrants and the majority population across age and sex. The data sources give us access to all the suicides in Norway between 2007 and 2014, as well as all contact with PHC. Thus, we enhance our understanding of healthcare use among suicide victims and whether there are differences between immigrants and the majority population. This may also be valuable information outside of Norway, since different perceptions of healthcare services and language barriers are challenges common to all countries with immigrant populations.

## Data and methods

This study is based on data covering the period 2006 to 2014 from the Norwegian Cause of Death Registry, the Norwegian Population Registry and the Database for the reimbursement of health expenses (KUHR). By means of unique personal identification numbers assigned to all Norwegian citizens and immigrants who stay over 6 months, we construct individual record linkages across the different data sources.

The data consists of 4341 suicide victims who died between 2007 and 2014, at age 15 years and older. People without a residence permit, e.g. asylum seekers living in reception centers and temporary guest workers not yet assigned unique personal identification numbers are not included in the study.

Information about the date and cause of death were obtained from the Norwegian Cause of Death Registry. Suicides are coded according to the ICD-10 codes X60-X84 and Y87.0 [16]. From the KUHR Database, we obtained information about dates of consultations with PHC, whereas information about immigration status, age and sex were obtained from the Norwegian Population Register.

The Regional Committee for Medical and Health Research Ethics granted ethical approval for the main study.

### Variables

The dataset is organized chronologically from 2007 to 2014. For the suicide victims who died early in 2007, we have also included contact with PHC made in 2006, to ensure that all victims have the same observation period prior to death.

Suicide victims’ age at death is grouped into following age ranges, 15–29, 30–44, 45–59, and 60+. Due to relatively few suicides in the oldest age group, we chose a low point of entry for this range.

Suicide victims’ immigrant status is categorized in a dichotomous variable. Foreign-born with foreign-born parents and Norwegian-born with foreign-born parents are classified as immigrants, while the rest is categorized as the majority population, where almost everyone is Norwegian-born with two Norwegian-born parents.

Time intervals between suicide and contact with PHC are categorized into: 6 months, 1 month, 1 week, and not in contact prior to suicide. One of the benefits of our data source is the possibility of very accurate estimations close to time of death. We also did calculations for contact with PHC within the year prior to suicide, but expanding the inclusion criteria did not add much value to the results. The rates were somewhat higher than those at 6 months, but closer in time to the suicide we can assume that the problems causing the suicide are more likely to be present. Cases with no contact within the last 6 months, but with contact more than 6 months prior to suicide, are grouped together with cases without contact between 2006 and 2014.

We defined “being in contact with PHC” as a personal consultation with a doctor or nurse. PHC in this study includes both the personal GP and other GPs at the local doctor’s office, and emergency rooms.

### Method

The analyses are descriptive, and the results are displayed in cross-tables. To investigate the differences in contact rates, we used the chi-square test for association. Analyses were performed using Stata 14.

## Results

In total, there were 4341 suicides in Norway between 2007 and 2014, of which approximately 70% were men. Immigrants accounted for less than 10% of the suicides, although immigrants aged 16 and over make up 15% of the Norwegian population [9, 10]. This means that immigrants’ suicide rate is lower than the rate for the majority population. Table 1 shows the percentage distribution of suicides by sex and age within each population group. About a quarter of the suicides in the majority population are committed by victims in the oldest age group. For both immigrant men and women, this age group has the fewest suicide victims. Among immigrants, suicides occur at a slightly younger age. Both immigrant men and women have higher percentages in the two youngest age groups than men and women in the majority population. Overall, the suicides are somewhat evenly distributed across the four age groups, but the highest occurrence of suicide overall is amongst the 45 to 59 year olds.

**Table 1:** Suicides in Norway 2007–2014

Age groups	Men		Women		Total
	Majority population	Immigrants	Majority population	Immigrants	
15–29	20% (566)	24% (64)	18% (206)	19% (19)	20% (855)
30–44	26% (743)	36% (98)	24% (280)	31% (31)	27% (1152)
45–59	27% (759)	27% (74)	33% (385)	33% (33)	29% (1251)
60+	26% (745)	13% (34)	25% (288)	16% (16)	25% (1083)
Total	100% (2813)	100% (270)	100% (1159)	100% (99)	100% (4341)

Suicide by sex, age and immigration status, percentages and number of suicides

### Contact with PHC prior to suicide

**Fig. 1:**
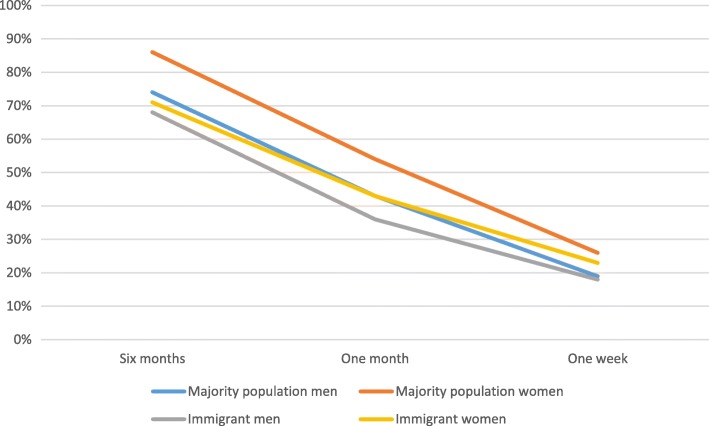
Use of primary healthcare services by sex and immigrant status. Percentage in contact 6 months, 1 month and 1 week prior to suicide, 2007–2014

Figure 1 shows the overall contact with PHC prior to suicide for the four groups: men and women in the majority population and immigrant men and women. Women in the majority population have consistently higher contact rates than the other groups, but the variance is minor 1 week prior to the suicide. Immigrant men have the lowest contact rate at all time periods, although there is practically no difference between the two male groups 1 week prior to suicide.

Overall, immigrant suicide victims are less in contact with PHC within 6 months prior to suicide, than majority population victims (χ2 18.8; d*f* 1). On average, immigrant men have a contact rate of 68%, while majority population men have 74%, and for women the equivalent rates are 71 and 86% (χ2 5.7; d*f* 1 for men and χ2 16.1; d*f* 1 for women). 1 month prior to suicide, the trend is similar, and is also statistically significant for both sexes (χ2 5.0; d*f* 1 for men and χ2 4.4; d*f* 1 for women). Immigrant men have a contact rate of 36%, while majority population men have 43%, whereas for women the equivalent rates are 43 and 54%. Within the last week prior to suicide, the differences are much less substantial (18 and 19% for men, and 23 and 26% for women). These differences are not statistically significant (χ2 0.4; d*f* 1 for men and χ2 0.4; d*f* 1 for women).

### Contact with PHC 6 months prior to suicide

The following paragraphs will show level of contact prior to suicide according to age, in addition to sex and immigrant status (Table 2).

**Table 2:** Contact with PHC within 6 months prior to suicide

6 months	Men		Women	
Age groups	Majority population	Immigrants	Majority population	Immigrants
15–29	62%	63%	79%	68%
30–44	73%	63%	86%	74%
45–59	76%	74%	87%	73%
60+	84%	76%	89%	63%
Average	74%	68%	86%	71%

Percentage in contact with PHC 6 months prior to suicide by sex, age and immigrant status, 2007–2014

Divided into age groups, it is only amongst the 45–59 and 60+ year olds that we find significant differences (χ2 5.4; d*f* 1 for 45–59 year olds and χ2 9.2; d*f* 1 for 60+ year olds), but merely for women. The contact rate increases with age for men in both groups. For immigrant women there is no clear age pattern, as the oldest and the youngest have the lowest rates of contact. Immigrant men and women have somewhat similar rates of contact with PHC within 6 months prior to suicide, while women in the majority population have significantly higher contact rates than men. Men aged 15–29 are the least in contact with PHC within 6 months prior to suicide, but there is no difference according to immigration status. Immigrant women in the same age range have a higher contact rate than the men, but lower than women in the majority population. We observe that the differences due to immigration status exceed differences due to sex.

### Contact with PHC 1 month prior to suicide

For both male and female suicide victims, the largest difference between the immigrant and the majority population 1 month prior to suicide is in the age group 30–44. However, the differences are only significant on a p-level of 0.1 (χ2 3.3; d*f* 1 for men and χ2 3.2; d*f* 1 for women). Among men, contact rates increase slightly with age, with the 60+ age group considerably higher than the rest. For women there is no clear pattern. Immigrant women have higher contact rates among the 15–29 and 45–59 year olds. As in the former table, the youngest suicide victims are the least in contact with PHC prior to suicide, except for the immigrant women. Still, men have substantially lower rates than do women. The oldest female immigrant suicide victims deviate from the other elderly, with much lower rates of contact. Overall, the immigrant differences are similar to the sex differences. Majority population female suicide victims stand out with substantially more contact with PHC 1 month prior to suicide than the three other groups (Table 3).

**Table 3:** Contact with PHC within 1 month prior to suicide

1 month	Men		Women	
Age groups	Majority population	Immigrants	Majority population	Immigrants
15–29	29%	27%	48%	47%
30–44	40%	30%	56%	39%
45–59	46%	43%	58%	52%
60+	52%	50%	53%	31%
Average	43%	36%	54%	43%

Percentage in contact with PHC 1 month prior to suicide by sex, age and immigrant status, 2007–2014

### Contact with PHC 1 week prior to suicide

The difference between immigrant and majority population men is much less substantial within the last week than 1 month prior to suicide. The same tendency is evident for women as well, but immigrant women still have lower contact rates 1 week prior to suicide than their majority population peers, for all ages except the 45–59 year olds. As seen above, the oldest immigrant women are less in contact with PHC prior to suicide than the other elderly. There is no clear age gradient, except with immigrant men. The youngest age group has the lowest contact rates within the last week prior to suicide. For the youngest immigrant men, the contact rate in the week prior to suicide is 14%, while for the majority population it is 16%. For women, the corresponding results are 16 and 25%. Among both immigrants and the majority population, women have a higher contact rate overall than men. The immigrant differences are lower than the sex differences, but the variation is minor (Table 4).

**Table 4:** Contact with PHC within 1 week prior to suicide

1 week	Men		Women	
Age groups	Majority population	Immigrants	Majority population	Immigrants
15–29	16%	14%	25%	16%
30–44	18%	17%	28%	26%
45–59	22%	19%	27%	30%
60+	21%	24%	24%	13%
Average	19%	18%	26%	23%

Percentage in contact with PHC 1 week prior to suicide by sex, age and immigrant status, 2007–2014

## Discussion

To our knowledge, this is the first overview of immigrants’ use of PHC prior to suicide. Generally, PHC usage is substantial for both groups, but immigrants are less likely to contact PHC prior to suicide than the population in general. This difference is evident for all the three time intervals examined, but it is most distinct 6 months and 1 month prior to suicide. In these time intervals, the estimates are statistically significant for both sexes.

The results confirm our main supposition: immigrants are less in contact with PHC prior to suicide than the majority population. In addition, the differences between immigrants and the majority population decreased closer in time to the suicide. This was evident for both sexes. This supports the hypothesis that increases in problems level out the differences between the populations. The differences between the immigrant and majority populations were also less substantial in the last week prior to suicide, when broken down by age. Both men and women aged 30–44 had large variances 6 months and 1 month prior to suicide, but these had nearly disappeared at the final time point. This age group comprised the largest percentage of both male and female immigrant suicide victims. This also lends support to the assertion that the difference between the groups decreases when the problems escalate. However, although we did not find significant differences within the last week prior to suicide, it is difficult to determine whether this was due to the paucity of observations or actual similarities between the groups.

The results presented here are at the average level found in international research on contact within the last 6 months and 1 month prior to suicide [4, 5]. The contact rates within the last week prior to suicide are higher in this study than previously found (total average 21.5% versus 16%). This holds regardless of immigrant status, sex and age, except for the youngest immigrant men. It is difficult to compare the difference between the majority and immigrant populations with other studies, since, to our knowledge, this is the first study to examine PHC use prior to suicide in an immigrant population.

Some groups stand out with noticeably lower contact rates than the rest, namely the youngest victims. For all three time periods, men aged 15–29 have contact rates well below average. For the victims in contact with PHC within the last month and/or the last week prior to suicide, immigrant men have even lower rates than majority population men. This is concerning because Table 1 shows that the age group 15–29 comprises 24% of the suicides committed by immigrant men, and 60% occur before the age of 45. The contact rate for both majority population and immigrant men increases with age. For female immigrant victims aged 15–29, the rate is low within the last 6 months and the last week prior to suicide compared to majority population women, but not within the last month prior to suicide. As suicide is one of the main causes of mortality in young people, and timely help can reduce suicide risk, there is, in our opinion, an unexploited potential for suicide prevention here. How to increase healthcare use among young (immigrant) individuals should be investigated.

Sex differences in contact with PHC prior to suicide is well established in the literature [5, 6]. From the results presented here, it seems that differences in contact due to immigrant status can be equally large, although it is difficult to draw a conclusion due to few significant findings, especially close in time to the suicide. For the PHC services to fulfill their suicide prevention potential there has to be contact between the victim and a healthcare professional. For instance there cannot be a referral to specialized healthcare services if the at-risk individual never contacts the PHC service.

The Norwegian healthcare system is publicly funded through the welfare state. Every resident is assigned a GP, and emergency rooms are available throughout the country. This means that healthcare services should be accessible at all times. From the results presented in this study, the propensity for contacting PHC prior to suicide is seemingly higher for the majority population than for immigrant victims. For immigrants, the barrier for help-seeking for mental health problems *may* be higher due to language difficulties or lack of knowledge about available health services [13]. According to a recent literature review of suicide risk among immigrants and ethnic minorities, lack of information on the healthcare system, as well as other migrant-related issues such as loss of status and/or social network, are possible triggers for suicidal behavior [17].

### Implications and future research

Contact with PHC prior to suicide is common in Norway, including shortly before suicide. There may be some unexploited potential for suicide prevention here, but more knowledge is required. To facilitate this, future research could focus on several aspects.

Firstly, research could focus on reason for contact. The current study contains all personal contact with PHC, but considering reason for contact could shed light on systematic differences between different types of medical problems. We know that the probability for contacting PHC for health problems is unevenly distributed in the population [13, 15, 18]. It would be valuable to know whether there are systematic differences between the majority and immigrant populations in the kind of health problems that lead to contact with PHC prior to suicide, because it could help identifying at-risk individuals.

Secondly, immigrant-relevant variables, such as country of origin, reason for migration and length of stay in Norway could affect both the suicide rate and the likelihood of seeking help prior to suicide. For example, Straiton and colleagues [18] reported that the usage of PHC for mental health problems is different for refugee and non-refugee immigrants. Diaz and colleagues [15] found that immigrants’ odds of contacting PHC vary with the wealth of their country of origin. Findings from the Oslo Health Study indicated that psychological distress is unevenly distributed among immigrants from high- and low-income countries [19]. When examining immigrant women’s use of PHC for mental health problems, Straiton and colleagues [18] emphasized that the use of the term “immigrants” obscures important differences in culture and health. Unevenly distributed chances of mental health problems and unevenly distributed chances of being in contact with PHC call for knowledge about who are more likely to be at risk, and who will contact PHC when in distress. In addition, increased knowledge will help in identifying the groups not in contact with PHC prior to suicide. Knowing more about who they are may help enable the healthcare services to make it easier for them to seek help. Thus, it could be advantageous for future research to break down the group by country of origin, reason for immigration or socioeconomic status.

Thirdly, future research could focus on the transition from institutions within the specialized healthcare services into PHC. The aftercare of people with mental health problems is organized within PHC. Increased utilization of inpatient treatment facilities is associated with higher suicide risk, and should thus prompt post-discharge treatment planning [20]. Reduction in care is associated with higher odds of suicide within 3 months of final contact [21]. If there are systematic differences in who gets better follow-up after inpatient care, this is something that the GPs and other healthcare professionals should be aware of. However, this requires more data sources than we have access to in this study.

Lastly, future research should investigate what kind of help is given to the individuals who contact PHC. It would be useful to know if there are any disparities between the help offered immigrants and the majority population, or systematic differences in who is referred to specialized healthcare. In the study regarding PHC use among immigrant and native Norwegian women, the results suggested some differences in use of psychotropic medicine and conversational therapy with the doctor. This may be linked to treatment preferences, cultural understanding or language difficulties [14]. It is only possible to make potential changes once the current practice is known.

### Strengths and limitations

The greatest advantage of this study is the access to high quality register data that includes the entire Norwegian population. Problems related to selection bias and attrition are thus minimal. The use of register data also allows the opportunity for measuring contact close to the time of suicide. Knowledge about help-seeking behavior close to suicide is valuable in order to increase suicide prevention, because the problems causing suicide risk are probably present so close in time.

These results include personal contact with the PHC system as a whole, meaning GPs and emergency rooms. The results would perhaps be easier to interpret if they specified contact points. It would then be clearer where efforts on suicide prevention could best be directed, and would say something about immigrants’ use of (different) primary healthcare services. In addition, there may be some differences in how the various contact points register their healthcare use, due to potential variations in registration procedures.

Suicide is a rare phenomenon, and even more so when considering the immigrant population specifically. This limits the study, as small numeric differences can seem large when read as percentages. Nonetheless, register data provides the best foundation for studying marginal phenomena, because it offers access to the entire population and a lack of attrition.

## Conclusion

Compared to international research, suicide victims in Norway contact PHC somewhat more than average, especially closer in time to the suicide. Victims with both immigrant and majority background follow the same overall pattern, with higher contact rates among women, although immigrants have a generally lower rate of contact than the majority population. The differences between the populations are more substantial 6 months and 1 month prior to suicide than within the last week. The youngest victims, and mainly the men, are least in contact with PHC prior to suicide. We find generally high use of PHC prior to suicide among the elderly, except for the oldest immigrant women. However, this is a small group, and it thus difficult to draw any conclusions. The differences due to immigrant status are seemingly equally as large as sex differences.

## Data Availability

The datasets generated and analyzed for the current study are not publicly available for data protection reasons.

## Abbreviations

GP: General practitioner
ICD: International classification of diseases
KUHR: Database for the reimbursement of health expenses
PHC: Primary healthcare
